# Providing healthy and climate-friendly public meals to senior citizens: a midway evaluation of a municipality’s food strategy

**DOI:** 10.1007/s00394-025-03728-4

**Published:** 2025-06-04

**Authors:** Anne Dahl Lassen, Matilda Nordman, Lene Møller Christensen, Ellen Trolle

**Affiliations:** 1 Research Group for Nutrition, Sustainability and Health Promotion, National Food Institute, Technical University of Denmark, Kgs Lyngby, DK-2800 Denmark; 2 Section for Quantitative Sustainability Assessment, Department of Environmental and Resource Engineering, Technical University of Denmark, Kgs Lyngby, DK-2800 Denmark

**Keywords:** Public food service, Dietary guidelines, Sustainable food procurement, Older adults

## Abstract

**Purpose:**

To evaluate changes in greenhouse gas emissions (GHGE) and nutritional composition of food procured for public meals for senior citizens from 2018 to 2022 in the city of Copenhagen. This provides a mid-way evaluation of the Food Strategy aimed at reducing the GHGE of public meals by 25% and assessing whether the municipality is on track to meet its reduction goals.

**Methods:**

Each food item purchased was matched with food composition and GHGE data, both with and without carbon opportunity costs. Data was analyzed separately for 2018, 2022, and both halves of 2022, as well as for different settings. Additionally, materials and initiatives provided by the City of Copenhagen were identified.

**Results:**

Across settings, a GHGE reduction of 14% (GHGE including carbon opportunity costs), and an 11% decrease in meat content was found from 2018 to 2022. Changes varied widely between settings with the largest reduction (30%) seen for the central kitchen producing mainly hot meals, while nursing homes providing full-day meals reduced GHGE by 10%. No changes were observed for the protein content, which did not meet the nutritional target for older adults. Municipal initiatives included meal guidelines and a recipe database provided in 2021. Additionally, by 2022, approx. one in five nursing homes, along with the central kitchen, had completed a tailored training program.

**Conclusion:**

The municipality is making progress towards meeting GHGE reduction goals for public meals for senior citizens. Further efforts are needed to increase pulses and other protein-rich plant-based products in daily meals.

**Supplementary Information:**

The online version contains supplementary material available at 10.1007/s00394-025-03728-4.

## Introduction

There is an urgent need to accelerate the transformation of our current food system towards one that is more sustainable for the planet and healthier for humans. Governments and municipalities have a unique opportunity to lead by example by ensuring that the food purchased and served in public settings contributes to sustainable consumption. This includes using more plant-based options such as vegetables, fruits, pulses, grains, nuts, seeds, as well as foods derived from these [1]. At the same time, public food service provides food to vulnerable groups, including older adults, and the nutritional quality must not be compromised.

Malnutrition predisposes older adults to an increased risk of adverse clinical outcomes such as frailty, osteoporosis, muscle wastage, and mortality [2]. Still, a newly published French cross-sectional study found that older adults’ energy and protein intake systematically fell short of requirements in both hospital and nursing-home settings [3]. The same picture has been observed in other studies, suggesting that low protein content is a prevalent concern in meals provided to older adults [4–6].

Experts and scientific advisory groups recommend a consumption of 1.0–1.2 g protein/kg body weight/d for adults over the age of 65 years to sustain muscle strength and function, and even greater amounts for individuals with severe illness or injury [7, 8]. According to the Nordic Nutrition Recommendations from 2023 for food planning purposes in older adults, a suitable target is 18 E%, corresponding to about 1.2 g protein/kg body weight/d [9].

Alongside these health based dietary recommendations among older adults, current sustainability concerns are driving a need for reduced consumption of foods with a high climate impact, such as beef and other animal sourced products [10, 11]. A transition towards a healthier plant- and protein-rich diet is a possibility for a win-win. Theoretical scenario analysis has shown that it is possible to obtain significant reductions in food-related greenhouse gas emissions (GHGE) and, at the same time, maintain or increase the protein content of the food served to older adults [12, 13]. However, research on the barriers and successes in implementing policy initiatives aimed at mitigating the climate impact of public food service is currently limited [14]. A qualitative study among procurement and food service officers in Danish municipalities and regions revealed a concern about the nutritional composition and acceptability of the meals for especially vulnerable older adults if changing to a more climate-friendly menu [15]. Van Wymelbeke et al. found that meal improvement strategies must take into account the organizational and economic constraints of the nursing home to ensure their implementation on a routine basis [16].

The adoption of strategies aimed at mitigating the climate impact of food procurement remains a relatively recent priority for city governments [15]. Nevertheless, in recent years, municipalities and cities worldwide have made commitments to work towards more healthy and sustainable food procurement with different areas of focus [17–20]. This applies, for example, to hospitals in New York City that have decided to serve culturally diverse, plant-based meals as primary dinner options at all of their public hospitals [21]. The city of Malmö, Sweden, is serving meals that are 70% organic, and has reported to have reduced their GHGE by 30% [22]. The Cool Food Pledge led by World Resources Institute (WRI) is committed to helping food-serving organizations and city governments to cut the climate impact of the food they serve by 25% by 2030 [23]. In 2019, the City of Copenhagen became a signatory of the Cool Food Pledge and launched a climate-friendly Food Strategy which came into force in 2020. The ambition is to reduce GHGE from public meals by 25% by 2025 relative to 2018 levels while simultaneously ensuring nutritional requirements, culinary quality and keeping organic food purchases at a high level [15].

The primary objective of this study was to evaluate the changes in GHGE and nutritional composition of food procured from 2018 to 2022 by the City of Copenhagen for public meals directed at senior citizens, including nursing home residents and senior citizens receiving home-delivered meals. A secondary objective was to examine differences between various types of nursing homes, including those providing full-day meals (all main and in-between meals) and those without in-house hot meal production, as well as food purchased by the municipality’s central kitchen (Københavns Madservice), and to analyse the differences between the first and second halves of 2022. Finally, the study aimed to identify materials and initiatives employed by the City of Copenhagen to fulfil the Food Strategy. This analysis serves as a mid-way evaluation of the Food Strategy implemented by the City of Copenhagen to reduce the GHGE of public meals, assessing whether the program is on track to meet its goals.

## Materials and methods

### Study design and settings

The study used a quasi-experimental research design (pre-post test) comparing 2018 and 2022 procurement data for public meals provided to senior citizens in the City of Copenhagen. The study represents a mid-way evaluation of the implementation of Copenhagen’s Food Strategy from 2019 which aims to reduce food-related GHGE by a minimum of 25% by 2025. The 25% reduction goal is based on GHGE data from WRI including both the agricultural supply chain emissions and carbon opportunity costs [23].

This study included three settings: a central kitchen; a total of 30 nursing homes providing full-day meals, i.e. all main and in-between meals; and 12 nursing homes without in-house hot meal production. The 12 nursing homes purchase food for breakfast, lunch, snacks (especially ready-made products) and dairy products while the hot meals are provided by the central kitchen. The central kitchen further delivers approx. 1,600 hot meals daily for senior citizens receiving home delivered meal services. To a smaller extent, the central kitchen also delivers meals to various social housing services, as well as food for receptions, etc.

Researchers were responsible for developing guidelines for healthy and climate-friendly meals to the nursing homes based on scenario modelling of procurement data in order to achieve the GHGE reduction goal and nutritional targets according to official dietary recommendations as described elsewhere [13]. Besides this, researchers solely played an observational role. Identification of materials and initiatives provided by the City of Copenhagen and initiatives implemented by the facilities was conducted through searches on the municipality’s official websites, document analysis [24–27] and two dialogue meetings with stakeholders, where the results were presented and progress discussed. These stakeholders included kitchen managers, health and care officers at the municipality and gastronomic consultants hired by the municipality.

### Data sources for procurement analysis 2018 and 2022

Data sources for nutrient and climate impact calculations included food procurement data for the City of Copenhagen in 2018 and 2022 comprising of food purchased from the main wholesale supplier as well as foods purchased from suppliers providing single food categories (e.g., fresh meat, fruit and vegetables). Food items from single food categories, which were recorded in price only, were converted into grams based on food-group specific kilo prices from the main wholesale supplier. Nutritional data were provided from the Danish Food Composition Database Frida (version 3) [28]. The Food Composition Table was checked for missing values on e.g. added sugar and missing data were either substituted with values sourced from the Swedish food composition Table [29] or with values obtained from comparable products.

Finally, climate impact data were derived from the WRI’s Cool Food Pledge calculator (version June 2022) [30]. Data on production-related emissions is based on European average values for GHGE from agricultural supply chains (termed Metric 2 by WRI), as presented by Poore and Nemecek [31]. The global average values for carbon opportunity costs (termed Metric 4 by WRI), have been outlined by Searchinger et al. [32], and quantify GHGE of global agricultural land use in terms of the amount of carbon lost from plants and soils as a result of agricultural expansion due to additional production of a food. In accordance to Searchinger et al. [32] it can be defined as the amount of carbon that could be stored if production of that food declined and land in agriculture returned to its native vegetation. Carbon opportunity cost has been shown to vary depending on the estimate method used [33, 34], and therefore, in line with WRI’s Cool Food Pledge, in the present study, GHGE is expressed both with and without carbon opportunity costs.

Estimation of nutritional content and GHGE were based on the edible amounts of the food products, e.g., excluding bones, peels, etc. To calculate edible amounts, estimated waste fractions were assigned to different foods as shown in the supplementary material of Lassen et al. [13].

### Procurement analysis

Data processing and analyses were carried out in R Statistics and Microsoft Excel. Each unique item in the food procurement data was matched with data from the food composition database and with climate impact data. When a good food match could not be found, data from similar foods, data from the product label or recipe data were used. Calculations of nutrient content and GHGE were performed on the pooled procurement data for each of the three settings in 2018 and 2022, respectively. The data were standardized to 10 MJ (approximately 2400 kcal) to improve comparability and assess nutrient content against guidelines.

### Comparison of the first and second halves of 2022

While some initiatives were provided to all in 2021, tailored training programs were planned to start at different times beginning in 2021 and continue through 2024. It was expected that changes in food procurement patterns would be more pronounced in the latter half of 2022 due to a higher proportion of kitchens having implemented changes following the tailored training program including education courses and workshops. Therefore, an analysis was conducted separately for the first and second half of 2022 to examine any potential differences between the two periods. Data for 2018 could not be divided into half-year periods.

## Results

### Background information

The food procured in the municipality for meals for senior citizens constituted approximately 3000 tons of food and beverages. This represents roughly one-third of the total food purchased by the municipality.

Food purchased by nursing homes providing full-day meals constituted approximately two-thirds of the total food purchased in the municipality for meals for senior citizens. Nursing homes without in-house hot meal production (where the hot meals are provided by the central kitchen) accounted for 15%, while the catering service supplying home delivered meal service to senior citizens and hot meals to nursing homes without in-house hot meal production accounted for 18% of the purchased food for meals for senior citizens (2022 data). Food purchased from the main wholesale supplier constituted 99.5% of food purchased by nursing homes providing full-day meals and 100% of food purchased by nursing home without in-house meal production. For the central kitchen, 66% and 59% of the food in 2018 and 2022, respectively, was purchased from the main wholesale supplier; the rest was provided by meat and fruit and vegetable suppliers.

### Materials and initiatives provided

Table 1 shows strategies, materials, and initiatives for the transition to healthy sustainable public meals provided by the City of Copenhagen at different time points with emphasis on initiatives especially relevant for GHGE reduction goals and nutritional quality. Initiatives aimed at reducing food waste are not described in the current paper but are also part of the municipality’s focus areas.

**Table 1:** Strategies, materials and initiatives provided to the kitchens by the City of Copenhagen to support the transition to healthy, sustainable public meals, aimed at achieving a 25% GHGE reduction in meals for senior citizens by 2025

Strategies, materials and initiatives	Core components	Key actors	Timeline nursing homes and central kitchen
The City of Copenhagen’s Food Strategy	The target is to implement a reduction in the GHGE of minimum 25% by 2025	The City of Copenhagen	2019 with start in 2020
Guidelines for healthy and climate-friendly meals	Guidelines for overall food composition and rules of thumb for climate-friendly and nutritious diets based on the official nutrition recommendations.	Provided to the staff by City of Copenhagen. Developed by researchers^a^ in dialogue with key actors consisting of gastronomic consultants for the municipality^b^ and key persons in the municipality.	Provided in 2021
Additional sustainability criteria into the food tender	Creation of a climate weighting procedure that reflects which products are important within the implementation of the municipality’s guidelines.	The City of Copenhagen	Included in 2020
Climate-friendly database of recipes	Recipes based on organic and seasonal foods, taking into account guidelines and taste and operational conditions of professional kitchens.	Gastronomic consultants^b^	2021
12-month tailored training program plus 3–6 month follow up, including quarterly climate reports as well as courses to support kitchen staff and the care personnel	Increase motivation and culinary skills among health professionals to help support implementation of the guidelines as well as individual feedback on climate impact status.	Gastronomic consultants^b^	2021 to 2024

^a^DTU National Food institute, ^b^ Employed by Meyers Madhus

In 2019, the City of Copenhagen’s Food Strategy was launched with the target of reducing GHGE by a minimum of 25% by 2025. There is an ongoing cooperation with WRI on the monitoring of the City of Copenhagen’s climate impact towards 2025 [26].

The dietary guidelines for meals targeting different settings included guidelines for overall food composition and rules of thumb for the number of servings over a one-week period for each main food group [13, 35]. These were sent by the municipality to all kitchens in fall 2021 together with information material on the strategy implementation process, including offering of supervision and courses in the municipality.

Also in 2021, additional sustainability criteria were included into the food tender, including the creation of a climate weighting procedure that reflects which products are important within the implementation of the municipality’s benchmarks and guidelines, e.g., more competitive prices for certain foods that the kitchens need to provide more of were weighted favorably, such as nuts. Additionally, in 2021, a recipe database for healthy and climate-friendly meals with more than 750 recipes was made public to all, and more recipes have been added regularly [36]. In 2023, a total of 1000 recipes were developed including 120 in-between meal recipes directed at both nutritionally at-risk senior citizens who are only able to eat small portions and residents without nutritional challenges [25].

Implementation of training and supervision to all nursing home kitchen staff by gastronomic consultants is ongoing from 2021 to 2024. Each nursing home has been offered a tailored training program lasting 12 months plus 3–6 month follow-up. The program is an integrated training program implementing the Food Strategy. The program typically includes a baseline analysis of the GHGE reduction potential, an action plan for reducing GHGE according to the guidelines, as well as follow-up visits together with quarterly climate reports on food purchases to assess the achievement of climate reduction goals. In addition to the training program, various courses have been offered to both the kitchen staff and care personnel [24] to increase motivation, skills and collaboration focusing on topics such as new climate-friendly meal recipes, the utilization of legumes, and communication across professions [37]. See Supplementary Table S1 for an overview of courses offered to nursing homes. Attendance was not mandatory for these courses.

Due to COVID-19, the start of the training programs for nursing homes was postponed to autumn 2021 [24]. Out of a total of 30 nursing homes providing full-day meals, 1 had finished the counselling process in 2021 and an additional 4 in 2022. In addition, seven nursing homes providing full-day meals started the counselling process in 2022. Among 12 nursing homes without in-house hot meal production, 3 finished the counselling process in 2022 and an additional 3 started up in 2022. In 2021, the central kitchen established a 1½-year plan that focused on gradually reducing the number of beef dishes, introducing more vegetarian options, and developing new dishes incorporating legumes and other plant-based protein sources. As a separate initiative, this effort also involved creating variations of traditional meat dishes, such as incorporating red lentils into stews. As part of the central kitchens training program, the central kitchen received quarterly climate reports regarding their food purchases. Finally, the central kitchen has on its own initiative participated in various development projects related to “green proteins”, exploring the utilization of quinoa, tempeh, and extruded legumes.

### Climate impact

**Fig. 1:**
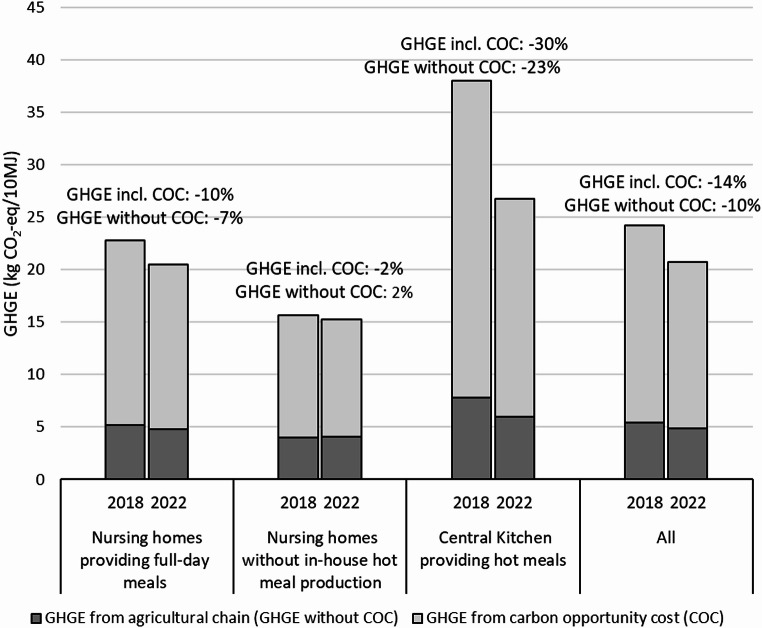
Estimated GHGE from the agricultural supply chains with carbon opportunity cost (GHGE including COC) and without carbon opportunity cost (GHGE without COC) for 2018 and 2022 for nursing homes providing full-day meals, nursing homes without in-house hot meal production and the central kitchen, respectively, and for all

From 2018 to 2022, the estimated food-related GHGE for nursing homes providing full-day meals decreased by 10% and 7%, with and without carbon opportunity costs, respectively, while corresponding reduction numbers were 30% and 23% for the central kitchen providing hot meals to nursing homes and to senior citizens receiving home delivered meals. In contrast, only small changes in GHGE values were seen for the nursing homes without in-house hot meal production (-2% and + 2% with and without carbon opportunity costs, respectively). Looking at the whole group together, the estimated food-related GHGE was reduced by 14% and 10%, respectively, with and without carbon opportunity costs (Fig. 1). The reduction in GHGE was primarily due to a decreased amount of ruminant meat. Reduction from ruminant meat alone was − 2.4 kg CO2e/10 MJ and − 12.1 kg CO2e/10 MJ, respectively, for nursing homes providing full-day meals and for the central kitchen providing hot meals (GHGE including carbon opportunity costs, data not shown).

### Nutrient content

Large differences in protein content were seen across different settings (Table 2). For 2018 data, the lowest protein content was seen in nursing homes without in-house hot meal production (11.5 E%), and highest for the central kitchen producing mainly hot meals (15.4 E%). In all cases, the protein content was below the recommended 18 E%. Fat content was high as recommended for the food served in Danish nursing homes due to a large proportion of nutritionally at-risk residents with poor appetite [38, 39]. The nutritional composition of the food procurement remained largely consistent from 2018 to 2022, with only minor alterations apparent during this period, including a small decrease in saturated fat content from 2018 to 2022 for the nursing homes without in-house hot meal production (18.6% to 17.2 E%) (Table 2). For comparison, the scenario target for the procurement for full-day meals in nursing homes is a maximum of 15 E% saturated fat as described by Lassen et al. [13]. This is for a combination of serving energy- and protein-dense diets directed at nutritionally at-risk residents and standard protein-dense diets for residents without nutritional problems. The recommendations for standard protein-dense diets alone are maximum 10 E% saturated fat [9].

**Table 2:** Content of macronutrients in food purchased by nursing homes providing full-day meals, nursing homes without in-house meal production and in the central kitchen (per 10 MJ) in 2018 and 2022, and comparison to recommendations [38]

Nutrients	Nursing homes								
	Providing full-day meals		Without in-house hot meal production		Central kitchen providing hot meals		All		
	2018	2022	2018	2022	2018	2022	2018	2022	Recom-mended density
Protein, total, g per 10 MJ	75	75	67	69	91	88	77	76	
Protein, total, E%	12.8	12.7	11.5	11.7	15.4	15.0	13.0	13.0	18^**a, b**^
Plant-based to animal-based protein ratio, %/%	28/72	30/70	33/67	32/68	28/72	33/67	29/71	32/68	
Carbohydrates, g per 10 MJ	226	229	253	259	231	233	231	234	
Carbohydrates, E%	40.0	40.4	44.7	45.6	41.1	41.6	40.9	41.4	
Added sugar, E%	11.8	11.9	14.9	16.1	9.2	9.1	11.8	12.1	
Fat total, g per 10 MJ	128	127	119	116	118	118	125	124	
Fat total, E%	47.3	47.0	44.0	42.8	43.6	43.5	46.2	45.8	40-50^**a**^/ 32–33^**b**^
Saturated fatty acids, E%	21.4	20.6	18.6	17.2	20.5	20.3	20.9	20.1	
n-3 fatty acids, E%	1.3	1.3	1.2	1.5	0.9	1.0	1.2	1.3	
Dietary fibre, g per 10 MJ	18	19	19	18	23	24	19	19	

^a^Recommended nutrient density according to the Danish official recommendations for nutritionally at-risk patients and senior citizens [38] ^b^Recommended nutrient density according for a standard protein-dense diet directed at residents without nutritional challenges according to the targets for diet planning for groups of individuals + 65 y from NNR [9]

A small increase in the protein contribution from plant-based foods to total protein was seen: on from 29 to 32% for all and more specifically from 28 to 30% for nursing homes providing full-day meals and from 28 to 33% for the central kitchen (Table 2).

In all instances, key essential amino acids associated with deficiencies, such as lysine and methionine, are present at levels well above the minimum WHO/FAO/UNU recommended density of 4.5 g/100 g protein and 1.6 g/100 g protein, respectively (see Supplementary Table S2).

### Changes in food procurement patterns

Table 3 presents the content of various foods purchased (per 10 MJ, edible fraction) by nursing homes providing full-day meals in 2018 and 2022. In addition, the table shows the target procurement amounts, which are based on modelled diets adhering to the Danish official recommendations for planning diets among institutionalized individuals [13]. The target is a combination of two main types of diets served by the nursing homes: an energy- and protein-dense diet provided to residents at risk of malnutrition (about 70% of the meals) and a standard protein-dense diet for those without nutritional challenges (about 30% of the meals). The high energy density required is reflected in the high content of cream, sour cream etc. and both animal and plant-based fats in both 2018 and 2022. However, there was a shift in fat composition, moving slightly away from cream, sour cream etc. and animal-based fats (-4% and − 7%, respectively) and towards an increased consumption of plant-based fats (+ 14%).

**Table 3:** Content of foods in 2018 and 2022 based on the municipality’s procurement data for nursing homes providing full-day meals and target for procurement amounts based on recommendation for meals served to institutionalized older adults

	Nursing homes providing full-day meals			
Food groups g per 10 MJ	2018	2022	Difference (%)	Target for food procurement (full-day’s meals)^a^
Bread and cereals^b^	142	148	4	140
Potatoes	122	109	-11	71
Vegetables, total^c^	137	141	3	128
Fruit, total^d^	107	111	0	118
Pulses, dry^e^	1.4	3.6	157	56
Processed plant-based protein-rich foods^f^	0.1	1.0	1115	-
Energy- and protein supplements	2.9	2.8	-1	-
Tree and ground nuts	1.2	1.2	-3	20
Seeds^g^	1.3	1.3	1	16
Milk	248	241	-3	248
Yoghurt etc.	82	101	24	95
Cream, sour cream etc.	71	68	-4	33
Cheese	34	35	3	23
Plant-based dairy alternatives	0.6	0.9	52	
Meat, total^h^	126	112	-11	96
Beef and lamb	36	26	-27	16
Pork	71	64	-9	61
Poultry	19	22	12	19
Egg	34	39	14	38
Fish, total^h^	44	42	-4	61
Fats, plant-based	23	27	14	25
Fats, animal-based^i^	33	31	-7	17
Discretionary foods and beverages	109	115	5	48
Condiments, seasoning, coffee and tea	21	22	3	20

^**a**^Based on scenario analysis reflecting nursing homes serving a combination of full-day energy- and protein-dense diets directed at nutritional at-risk residents (estimated to be about 70% of the diets provided) and the rest standard protein-dense diets directed at residents without nutritional challenges [13] according to the Danish official recommendations for dietary planning among institutionalized people [38]; ^b^Combination of grains/flour and bread; ^c^Includes mushrooms; ^d^Includes berries, dried fruit and fruit juice; ^e^ Pulses are here expressed as dry weight but is modelled as a combination of 48 g cooked pulses and 36 g pulses flour or protein powder; ^f^Soy-, pea-, and mycoprotein-based products, including tofu, plant-based nuggets, sausages etc.; ^g^Does not include seeds in bread; ^h^Meat and fish is predominantly unprocessed but also contains limited amounts of processed products; ^i^Includes also fat-based products e.g. sauces and dressings

Aligning well with the target amounts, no substantial changes in the content of bread and cereals, fruit, and vegetables per 10 MJ were observed from 2018 to 2022 among nursing homes providing full-day meals. On the other hand, there was a decrease in the overall amount of meat purchased (-11%), particularly ruminant meat (-27%), but also, to a lesser extent, a reduction in pork (-9%), while poultry and egg content increased by 12% and 14%, respectively. Thereby, the nursing homes providing full-day meals are getting closer to achieving the goals for the amount of meat. Moreover, pulses increased considerably in relative terms (+ 157%), but the content is still far from the target, as initial values were low. Nut, seed and fish content only changed to a minor extent,

Table 4 shows amounts of different foods procured per 10 MJ (edible fraction) by nursing homes without in-house hot meal production and by the central kitchen in 2018 and 2022. The percentage reduction of total meat from 2018 to 2022 was on the same level for nursing homes without in-house hot meal production as it was for the central kitchen (14% and 11%, respectively). However, the baseline amount of meat per 10 MJ was almost four times higher for the central kitchen providing hot meals compared with nursing homes without in-house hot meal production, and the amount of ruminant meat, which has one of the highest climate footprints, was approximately six times higher at baseline.

**Table 4:** Content of foods in 2018 and 2022 based on the municipality’s procurement data for nursing homes without in-house hot meal production and the central kitchen

Food groups per 10 MJ	Nursing homes without in-house hot meal production			Central kitchen providing hot meals		
	2018	2022	Difference (%)	2018	2022	Diffe-rence (%)
Bread and cereals^a^	195	189	-3	82	89	8
Potatoes	33	21	-37	325	338	4
Vegetables, total^b^	62	62	0	394	393	0
Fruit, total^c^	136	153	13	112	106	-5
Pulses, dry^d^	0.3	0.4	30	4.2	7.8	86
Processed plant-based protein-rich foods^e^	0.1	0.4	372	0.4	0.9	108
Energy- and protein supplements	4.0	4.9	23	0.1	0.2	106
Tree and ground nuts	0.9	0.6	-28	1.8	2.2	19
Seeds^f^	0.6	0.5	-20	0.3	0.8	176
Milk	280	267	-5	154	155	0
Yoghurt etc.	128	146	14	24	21	-11
Cream, sour cream etc.	47	40	-16	122	123	1
Cheese	51	48	-5	5.7	7.3	28
Plant-based dairy alternatives	0.0	1.9	40,128	0.4	1	114
Meat total^g^	60	51	-14	251	222	-11
Beef and lamb	8.9	8.1	-9	102	56	-46
Pork	39	33	-16	100	118	19
Poultry	12	10	-13	48	48	-1
Egg	25	36	41	20	25	23
Fish, total^g^	38	52	35	51	45	-12
Fats, plant-based	20	36	82	14	17	20
Fats, animal-based^h^	35	23	-33	15	13	-14
Discretionary foods and beverages	157	181	15	70	70	1
Condiments, seasoning, coffee and tea	12	19	58	30	34	12

^a^Combination of grains/flour and bread; ^b^Includes mushrooms; ^c^Includes berries, dried fruit and fruit juice; ^d^Pulses purchased as a mix of cooked and dry pulses but are here expressed as dry weight; ^e^Soy-, pea-, and mycoprotein-based products, including tofu, plant-based nuggets, sausages etc.; ^f^Does not include seeds in bread; ^g^Meat and fish are predominantly unprocessed but also contain limited amounts of processed products; ^h^Includes also fat-based products e.g. sauces and dressings

Compared with the 2018 level, the central kitchen reduced their purchase of ruminant meat by 46 g per 10 MJ, corresponding to a 56% decrease. Also, a reduction in fish content was seen by 6 g (-12%) while pork increased by 18 g (19%) and egg by 5 g per 10 MJ (23%). There was almost a doubling of pulses and processed plant-based protein-rich food for the central kitchen, but the initial level was considerably lower than meat (total increase of 4 g per 10 MJ). In addition, nuts and seeds increased slightly by 0.8 g per 10 MJ in total.

Nursing homes without in-house hot meal production moderately decreased the procurement of pork and poultry, while simultaneously increasing the quantities of eggs and fish. In relation to pulses, nuts, and seeds, only very small changes in absolute content were observed.

There was a shift in fat composition for both nursing homes without in-house hot meal production and the central kitchen, moving away from animal-based fats (-33% and − 14%) towards an increased consumption of plant-based fats (+ 81% and + 20%). On the other hand, amounts of cream, sour cream etc. remained high, with only a small decrease for nursing homes without in-house hot meal production (-16%).

Supplementary Table S3 shows amounts of different foods procured per 10 MJ for all settings together providing meals to senior citizens in the municipality. The decrease in meat content was 11%, particularly through a decrease in beef content (-34%), whereas increases were seen for eggs (18%) and yoghurt (20%) and for plant-based protein-rich foods such as pulses (126%).

### Comparison of the first and second halves of 2022

Supplementary Table S4 shows analysis conducted separately for the food procured during the first and second halves of 2022 and the difference between the half years. For all settings together, GHGE differed by -10% and − 7% with and without carbon opportunity costs, respectively, from first to second half year of 2022. When comparing the second half of 2022 with the entire year of 2018, the changes for all settings combined were − 19% and − 13%, with and without carbon opportunity cost, respectively (not shown).

The largest differences in GHGE between the two halves of 2022 were observed for the central kitchen, with reductions of -15% and − 11% with and without carbon opportunity costs, respectively. Reductions of the same magnitude were seen for nursing homes providing full-day meals (-9% and − 6% with and without carbon opportunity costs, respectively) and for nursing homes without in-house meal production (-8% and − 6%, with and without carbon opportunity costs, respectively).

The protein content in all nursing homes remained constant from first to second half year.

Supplementary Table S5 shows amounts of different foods procured per 10 MJ (edible fraction) separately for the first and second halves of 2022 for the different settings. The reductions in GHGE for all settings are reflected by especially a decrease in ruminant meat from the first to the second half of 2022: i.e. 23%, 20% and 29%, respectively, for nursing homes providing full-day meals, nursing homes without in-house hot meal production and the central kitchen (GHGE including carbon opportunity costs). The content of pulses increased from the first to the second half of 2022 only for nursing homes providing full-day meals and the central kitchen (15% and 13%, respectively).

## Discussion

The present study represents a mid-way evaluation of the implementation of the Food Strategy by the City of Copenhagen, which has the goal of achieving a 25% reduction in the climate impact, GHGE including carbon opportunity costs, of food procurement by 2025 compared with the baseline year of 2018. Based on 2022 data, a GHGE reduction of 14% and 10% with and without carbon opportunity costs, respectively, was observed across all settings providing meals for senior citizens in the municipality. Overall, the findings indicate that the municipality is on track to meet the 2025 climate impact reduction target of 25%, including carbon opportunity costs, assuming that the current rate of reduction continues. Municipal initiatives, such as meal guidelines and a recipe database provided in 2021, have been implemented. However, since only one out of five nursing homes, aside from the central kitchen, completed their training program in 2022, a further decrease is reasonable to expect. The nutritional composition of the procured food remained largely consistent, with no observed change in protein content. Conversely, the protein content did not increase, and therefore still did not meet recommendations set by dietary guidelines for older adults [9].

The reduction in GHGE observed in this study of 14%, including carbon opportunity costs, is comparable with climate reduction reports from The Cool Food Pledge, which is based on results from 48 members who joined prior to 2022 encompassing cities, companies, healthcare facilities, restaurants, and universities. They achieved an overall 10% reduction in per-plate emissions compared with their respective base years (2015–2018) when similarly including carbon opportunity costs. Notably, healthcare facilities alone demonstrated a reduction rate of 21% [40]. There is no reporting on the effects on the nutritional content.

In the present study, a large variation in GHGE was observed across different settings that provide meals to senior citizens in the municipality. The most substantial reduction in GHGE was identified in the central kitchen, providing hot meals, showing a reduction at 30% and 23% with and without accounting for carbon opportunity costs, respectively. Among nursing homes providing full-day meals– constituting a third of the food purchased for senior citizens in the municipality– a decrease of 10% and 7%, respectively, was observed with and without accounting for carbon opportunity costs. Only minor changes in GHGE were found over the investigated years for nursing homes without in-house hot meal production.

The large differences in GHGE reduction are likely, at least in part, due to different initial climate impact levels resulting from the preparation of different meal types and variations in the quantity of meat procured. This means that it will be easier for kitchens preparing hot meals to reduce GHGE by both decreasing the amount of meat served and adjusting the relative amounts of different types of meat, such as using a lower proportion of beef relative to other meat types, as seen in the present study. This also indicates that not all settings should be treated equally in terms of percentage reduction goals. To achieve an overall 25% reduction goal, some settings will need to contribute a higher reduction compared to others. Moreover, variations in the extent of implementation of the counselling program provided by the municipality, which is an ongoing initiative leading up to 2025, would also likely contribute to the observed differences in GHGE reduction. The comparison between the first and second halves of 2022 provides support for the assumption that alterations in food procurement patterns would be more pronounced in the latter half of 2022 due to higher number of kitchens having implemented changes following individual training and supervision. When comparing the second half of 2022 with the entire year of 2018, the overall change across all settings was a decrease of 19% GHGE including carbon opportunity costs. The central kitchen, on its own initiative, participated in various development projects related to “green protein”. This may complicate the ability to assess how the program under study is working. On the other hand, it could be argued that these additional initiatives were likely adopted as a spin-off of the municipality’s focus on climate reduction.

The GHGE reduction in this study was primarily attributed to a decrease in the purchase of beef. In nursing homes providing full-day meals and the central kitchen providing hot meals, the reduction in purchased ruminant meat was 27% and 46%, respectively, compared with the baseline in 2018. For comparison, the Cool Food Pledge reported a 28% decrease in the share of beef and lamb on the plate for healthcare facilities based on data from 23 members [40]. The same picture with reduction of ruminant meat as the main driver of climate impact reduction has been found in other settings, including a study evaluating changes in food procurement in childcare centres in the City of Copenhagen [41]. Additionally, Lambrecht et al. suggest that there is considerable potential for reducing food-based GHGE emissions in higher education institutions through replacing meat, particularly beef, with lower-emission animal and plant proteins, however the study did not measure the nutritional impacts of the proposed substitution scenarios [42]. Tregear et al. conclude that adjusting menu compositions, particularly by reducing the proportion of ruminant meat, is an important action for lowering emissions in primary schools [43].

In addition to the reduction in total meat, particularly through a decrease in beef content, some changes were also observed by transitioning to other animal-based products with less GHGE impact, such as eggs (+ 18% for all) and yoghurt etc. (+ 20% for all). Moreover, fish and seafood have demonstrated a range of beneficial health effects such as protection from cognitive decline in adults and providing nutrients such as n-3 fatty acids, iodine, selenium and protein. However, considering environmental impacts, priority should be given to fish and seafood from sustainably managed farms and wild stocks, while consumption of species with high environmental impact should be limited [9].

The inclusion of pulses increased considerably in relative terms, however the content is still far below the target, given the initially low values. Introducing pulses into meals for residents in nursing homes may present challenges. The residents in nursing homes are, on average, about 81 years old when they move in [44] and aspects like familiarity and recognition in relation to acceptance of meals has been found to be crucial (Okkels m.fl. 2019). Thus, the residents may be less inclined to try new dishes compared with younger individuals [45, 46]. Yet, Svendsen et al. suggest that some novel culinary “twists” may be acceptable and could stimulate food intake by preventing boredom [47].

Additionally, it may pose challenges for kitchen staff, requiring new skills and additional work processes, such as incorporating pulses into minced meat dishes, sauces, baked products, in-between meals, and drinks. In the present study, no measurements of barriers or staff acceptability were conducted. However, two dialogue meetings held to present the results of this study to e.g. kitchen managers and gastronomic consultants also revealed difficulties such as the need for more time in developing and testing recipes, including seasoning pulses, and the potential requirement for new equipment.

Concerning nuts and seeds, only very small changes in content were observed. This does not align with the guidelines and should be a clearer focus area in future initiatives. Nuts, along with pulses, are valuable sources of protein, dietary fiber, various micronutrients and other bioactive compounds, and nuts and seeds are also high in unsaturated fatty acids [9, 48, 49]. A recent prospective cohort study found that nut consumption was strongly associated with a lower risk of frailty among older women, after adjusting for health and lifestyle factors [50]. Simultaneously, staff should be aware of potential allergy risks within the population.

Across settings, there was a small increase in the protein contribution from plant-based foods to total protein: from 29 to 32%. While this is still below the calculated target of 46%, these changes represent a step in the right direction. A study across four European countries found that 37% of the total protein consumed by older individuals aged 65–79 years was plant-derived. The study concluded that alteration of the protein source distribution in favour of plant-derived proteins would be feasible with safe margins against deficiencies in essential amino acids [51]. Similarly, the current study indicates the provision of an adequate ratio of all essential amino acids, due to the content of animal-based protein as well as a variety of plant-based sources, including pulses and cereals that together provide all the essential amino acids [52]. Furthermore, the protein intake pattern throughout the day, in addition to total protein intake, has been suggested as an important factor for protein turnover and muscle mass [53]. Particularly, stimulating a higher protein intake at breakfast and snacks and/or finger foods seems to be an underutilized area and might represent a promising nutritional strategy for increasing protein content per meal. In the present study, the food procured by the nursing homes without hot meal production had the lowest protein content. Higher protein intake at breakfast and lunch has been associated with a higher total daily protein intake in community-dwelling older adults [54]. Likewise, another study showed that serving targeted high-quality protein-enriched breakfast improved patients’ protein- and energy intake (mean age 69 y) at breakfast as well as total daily protein- and energy intake [55].

In accordance with recommendations, a shift in fat composition moving away from animal-based fats towards an increased consumption of plant-based fats was seen in all settings. This is reflected in a small decrease in saturated fat E% for the nursing homes without in-house hot meal production. On the other hand, the amounts of cream, sour cream etc. remained high compared with the target, although a decrease of 16% was observed for nursing homes without in-house hot meal production.

This study indicates the important role of clear GHGE goals and supporting activities in effectively reducing GHGE levels across public food service. The municipal initiatives included providing guidelines for food composition and establishing rules of thumb for the number of servings per week for each main food group [13, 56]. Additional initiatives involved incorporating sustainability criteria into food tenders, creating a recipe database for healthy and climate-friendly meals, and implementing ongoing training and supervision for all nursing home kitchen staff by gastronomic consultants, scheduled from 2021 to 2024, with the provision of climate reports regarding their food purchases. Guillaumie et al. emphasize the necessity for guidance through training and recommendations in study programs targeting both future healthcare professionals and patients [57]. Furthermore, the implementation of an easy-to-use monitoring and documentation system is crucial for measuring compliance and progress towards sustainable practices [15, 58, 59]. Likewise, it is important to ensure that unintended consequences, such as impacts on palatability and nutritional content of meals, are carefully addressed and avoided. Conducting taste tests among nursing home residents and senior citizens receiving home-delivered meal services were not part of the present study. However, the municipality’s initiatives included focus on improving culinary skills among health professionals, reducing food waste, and providing recipes that align with senior citizens’ taste preferences.

This study possesses both strengths and limitations. The study is unique in examining a large-scale implementation of climate-friendly public food service within a real-life context. Other strengths of the study lie in the assessment of both the climate and nutritional impacts of the implementation and the utilization of procurement data, enabling an objective evaluation that is further segmented into various settings where public meals to senior citizens are provided. Technological advancements now enable the systematic collection of purchase data, offering detailed and precise information on the utilized food products. However, there are inherent limitations of the method, including its inability to differentiate between foods used for various purposes, such as different types of meals. Additionally, the data only reflect the food purchased and potentially served, without accounting for actual consumption and waste [60, 61]. Concurrently, efforts in the municipality’s kitchens have been underway to reduce food waste. Finally, one of the limitations of the method is potential missing data such as weight per unit, particularly from smaller individual suppliers. To address this, for products documented solely by price, a conversion into grams was conducted using food-group-specific kilo prices obtained from the primary wholesale source. Further, to ensure accurate categorization, each food item was linked to a proxy food item and coded into a specific food category by the researchers. Still, imprecision may arise from the food composition and climate impact databases containing generic data on foods, as opposed to product-specific data.

The study adopted an uncontrolled before-and-after design, precluding the establishment of a causal link between the intervention and the observed changes. Moreover, due to the impact of COVID-19, the start of the counseling process for the nursing homes was delayed until autumn 2021. Consequently, fewer kitchens had completed the training program at this mid-way analysis compared with the original plan.

The climate impact data available carries significant uncertainties, and the chosen approach has the potential to markedly influence the results [62]. The carbon opportunity costs utilized in this study, derived from WRI data, represent estimates for each food on how changes in the output types, output quantities and production processes of a hectare of land contribute to the global capacity to store carbon and to reduce total GHGE [32]. It comprises a different approach to incorporating estimates related to changes in land use, which may be relatively higher compared to other estimates that incorporate direct or indirect land use change [34]. Therefore, results on GHGE reduction in the present study are expressed both with and without carbon opportunity costs. Finally, more environmental footprints as well as food waste should be considered for more comprehensive evaluation of the environmental consequences of the observed shifts in food procurement.

## Conclusion

The present study contributes to the growing body of literature addressing the governance of healthy and sustainable food transitions. It offers a mid-way evaluation of the City of Copenhagen’s Food Strategy for providing healthy and climate-friendly public meals to senior citizens. Initiatives from the municipality involved guidelines for food composition, a recipe database, tailored training programs for kitchen and care staff as well as additional sustainability criteria in the food tender. Analysis of procurement data from 2018 to 2022 suggests that the the municipality is on the right path, demonstrating a GHGE decrease of 14% and 10% with and without carbon opportunity costs. There is, however, a notable variation depending on the setting and the types of meals being produced. While there is still progress to be made for full implementation, the City of Copenhagen serves as a promising model for other municipalities and policymakers aiming to transition toward more plant-rich food procurement. Progress toward the 2025 target will be monitored within the WRI Cool Food Pledge program.

The results highlight the importance of maintaining an ongoing focus on nutritional quality, particularly addressing the prevalent concern of too low protein content in meals provided to senior citizens. Therefore, alongside reducing total meat purchases, particularly ruminant meat, it is essential to incorporate a significantly higher proportion of protein-rich foods, such as pulses, nuts and seeds in both main meals, snacks and protein drinks to increase overall protein content in line with recommendations. Further research is required addressing environmental and nutritional goals synergistically. Additionally, future research should explore the impact of optimized climate-friendly diets on acceptability, actual intake, nutritional status, and quality of life for older adults.

## Electronic supplementary material

Below is the link to the electronic supplementary material.

Supplementary Material 1

## Data Availability

The data that support the findings of this study were provided by the City of Copenhagen, but restrictions apply to the availability of these data, which were used with permission for the current study and thus are not publicly available. In case of further interest in these data, requests must be made to the City of Copenhagen’s Budget Team in the Children and Youth Administration.
