# Supplementary material for: Providing healthy and climate-friendly public meals to senior citizens: a midway evaluation of a municipality’s food strategy
Source: Eur J Nutr. 2025 Jun 4;64(5):204. doi: 10.1007/s00394-025-03728-4 (PMC12137513; doi:10.1007/s00394-025-03728-4)
Supplement: Supplementary file 1 — Supplementary Material 1 [file 394_2025_3728_MOESM1_ESM.docx]

**supplementary material**

**PROVIDING HEALTHY AND CLIMATE-FRIENDLY PUBLIC MEALS TO SENIOR CITIZENS: A MIDWAY EVALUATION OF A MUNICIPALITY’S FOOD STRATEGY**

The European Journal of Nutrition

Anne Dahl Lassen, Matilda Nordman, Lene Møller Christensen, Ellen Trolle

Research Group for Nutrition, Sustainability and Health Promotion, National Food Institute, Technical University of Denmark, DK-2800 Kgs Lyngby

Corresponding author: [adla@food.dtu.dk](mailto:adla@food.dtu.dk)

**Table S1** Overview of courses offered that nursing homes can enroll in [1]

| **Courses and workshop description** | **Target group** |
| --- | --- |
| Course in classic dishes with a greener twist | Kitchen staff |
| Inspiration tour to an organic farm | Entire staff group |
| Course on the use of legumes and which pulses are good for what | Kitchen staff |
| Presentation and consultation on guidelines and climate reports, including reducing the amount of meat by adding more plant based proteins and reducing butter and cream and replacing, for example with oils | Kitchen staff |
| Course in baking bread and tarts with a climate-friendly perspective incorporating pulses | Kitchen staff |
| Presentation or workshop on nutritionally balanced meals and snack meals as a shared responsibility | Kitchen and care staff |
| Course in good basic recipes for homemade cakes | Kitchen and care staff |
| Meeting on purchasing analysis with a focus on organic, economics, climate and nutrition | Kitchen staff and Procurement manager |
| Brief presentation on climate adaptation for the older adults for the entire staff | care staff |
| Course in snack meals and the use of new recipes | Kitchen and care staff with food responsibility |
| Workshop on reducing food waste from department to kitchen | Entire staff group |
| Workshop on hospitality for meals | Entire staff group |
| Course in salads with pulses | Kitchen staff |
| Workshop on collaboration and communication across professions with a focus on the individual nutritional needs and preferences of the older adults | Entire staff group |

**Table S2** Essential amino acids content (g/100 g protein) in the food procurement in nursing homes both with and without in-house meal production and in the central kitchen (per 10 MJ) in 2018 and 2022, and comparison to recommended density

|  | **Nursing homes** | | | | | | | | | | | |  |  |  |
| --- | --- | --- | --- | --- | --- | --- | --- | --- | --- | --- | --- | --- | --- | --- | --- |
|  | **Providing full-day meals** | |  | | **Without in-house**  **hot meal production** | | | | | **Central kitchen providing hot meals** | | | |  |  |
| **Essential amino acids**  **g/100 g protein** | **2018** | **2022** |  |  | |  |  | **2018** | **2022** | | **2018** | **2022** | | | **Recommended density ^1^** |
| Isoleucine | 4.5 | 4.6 |  |  | |  |  | 4.7 | 4.7 | | 4.4 | 4.4 | | | 3.0 |
| Leucine | 7.5 | 7.5 |  |  | |  |  | 7.9 | 7.9 | | 7.0 | 7.0 | | | 5.9 |
| Lysine | 6.7 | 6.7 |  |  | |  |  | 6.5 | 6.5 | | 7.2 | 7.1 | | | 4.5 |
| Methionine | 2.2 | 2.2 |  |  | |  |  | 2.2 | 2.3 | | 2.2 | 2.1 | | | 1.6 |
| Phenylalanin | 4.3 | 4.3 |  |  | |  |  | 4.6 | 4.6 | | 3.9 | 3.9 | | | 3.8 |
| Threonine | 3.6 | 3.6 |  |  | |  |  | 3.6 | 3.6 | | 3.7 | 3.7 | | | 2.3 |
| Tryptofan | 1.2 | 1.2 |  |  | |  |  | 1.2 | 1.2 | | 1.1 | 1.1 | | | 0.6 |
| Valine | 5.5 | 5.5 |  |  | |  |  | 5.8 | 5.8 | | 5.3 | 5.3 | | | 3.9 |
| Histidine | 2.5 | 2.5 |  |  | |  |  | 2.5 | 2.5 | | 2.6 | 2.6 | | | 1.5 |

WHO/FAO/UNU for adults [2]

**Table S3** Content of foods g per 10 MJ for all settings providing food to older adults in the municipality based on procurement data

|  | **All settings providing foods to older adults** | | |
| --- | --- | --- | --- |
| **Food group g per 10 MJ** | **2018** | **2022** | **Difference**  **(%)** |
| Bread and cereals ^1^ | 140 | 145 | 3% |
| Potatoes | 142 | 133 | -6% |
| Vegetables, total ^2^ | 167 | 170 | 2% |
| Fruit, total ^3^ | 112 | 116 | 3% |
| Pulses, dry ^4^ | 1.7 | 3.8 | 126% |
| Processed plant-based protein-rich foods ^5^ | 0.1 | 0.9 | 552% |
| Energy- and protein supplements | 2.6 | 2.7 | 5% |
| Tree and ground nuts | 1.3 | 1.3 | 0% |
| Seeds ^6^ | 1.0 | 1.1 | 7% |
| Milk | 238 | 231 | -3% |
| Yoghurt etc. | 79 | 95 | 20% |
| Cream, sour cream etc. | 75 | 73 | -4% |
| Cheese | 32 | 32 | 2% |
| Plant-based dairy alternatives | 0.5 | 1.1 | 119% |
| Meat total | 136 | 121 | -11% |
| Beef and lamb | 43 | 28 | -34% |
| Pork | 71 | 68 | -3% |
| Poultry | 23 | 24 | 6% |
| Egg | 30 | 36 | 18% |
| Fish, total ^7^ | 44 | 44 | -1% |
| Fats, plant-based | 21 | 26 | 24% |
| Fats, animal-based ^8^ | 30 | 27 | -12% |
| Discretionary foods and beverages | 110 | 117 | 7% |
| Condiments, seasoning, coffee and tea | 21 | 23 | 10% |

^1^ Combination of grains/flour and bread; ^2^ Includes mushrooms; ^3^ Includes berries, dried fruit and fruit juice; ^4^ Pulses purchased as a mix of cooked and dry pulses but are here expressed as dry weight; ^5^ Soy-, pea-, and mycoprotein-based products, including tofu, plant-based nuggets, sausages etc.; ^6^ Does not include seeds in bread; ^7^ Meat and fish is predominantly raw but also contains limited amounts of processed products; ^8^ Includes also fat-based products e.g. sauces and dressings

**Table S4** Estimated GHGE (kg CO2e/10 MJ) from total agricultural supply chains (GHGE without COC) and combined total agricultural supply chains and carbon opportunity costs (GHGE including COC) for 1^st^ and 2^nd^ half of 2022, respectively, based on the municipality’s procurement data

|  | **Nursing homes** | | | | | |  | |  |
| --- | --- | --- | --- | --- | --- | --- | --- | --- | --- |
|  | **Providing full-day meals** | | | **Without in-house**  **hot meal production** | | | **Central kitchen**  **providing hot meals** | | |
|  | **2022**  **1^st^ half** | **2022**  **2^nd^ half** | **Diff 1^st^ and 2^nd^ half 2022 (%)** | **2022  1^st^ half** | **2022 2^nd^ half** | **Diff 1^st^ and 2^nd^ half 2022 (%)** | **2022  1^st^ half** | **2022 2^nd^ half** | **Diff 1^st^ and 2^nd^ half 2022 (%)** |
| GHGE without COC (kg CO_2_-e) | 5.0 | 4.7 | -6% | 4.2 | 3.9 | -6% | 6.3 | 5.6 | -11% |
| GHGE incl. COC (kg CO_2_-e) | 21.4 | 20.0 | -9% | 15.9 | 14.6 | -8% | 29.0 | 24.6 | -15% |

**Table S5** Content of foods g per 10 MJ in 1^st^ and 2^nd^ half of 2022, respectively, based on the municipality’s procurement data

|  |  | **Nursing homes** | | | | |  |  |  |
| --- | --- | --- | --- | --- | --- | --- | --- | --- | --- |
|  | **Providing full-day meals** | | | **Without in-house**  **hot meal production** | | | **Central kitchen providing hot meals** | |  |
| **Food group g per 10 MJ** | **2022**  **1^st^ half** | **2022**  **2^nd^ half** | **Diff 1^st^ and 2^nd^ half 2022 (%)** | **2022  1^st^ half** | **2022 2^nd^ half** | **Diff 1^st^ and 2^nd^ half 2022 (%)** | **2022  1^st^ half** | **2022 2^nd^ half** | **Diff 1^st^ and 2^nd^ half 2022 (%)** |
| Bread and cereals ^1^ | 149 | 148 | -1 | 188 | 189 | 0 | 92 | 87 | -6 |
| Potatoes | 109 | 109 | 0 | 22 | 19 | -11 | 324 | 351 | 8 |
| Vegetables, total ^2^ | 142 | 141 | -1 | 63 | 60 | -5 | 388 | 397 | 2 |
| Fruit, total ^3^ | 110 | 108 | -2 | 148 | 157 | 6 | 107 | 102 | -4 |
| Pulses, dry ^4^ | 3.3 | 3.9 | 15 | 0.4 | 0.3 | -19 | 7.3 | 8.3 | 13 |
| Processed plant-based protein-rich foods ^5^ | 0.8 | 1.2 | 46 | 0.3 | 0.5 | 47 | 0.5 | 1.3 | 145 |
| Energy- and protein supplements | 3.0 | 2.6 | -13 | 5.6 | 4.3 | -23 | 0.2 | 0.2 | 33 |
| Tree and ground nuts | 1.1 | 1.3 | 19 | 0.4 | 0.9 | 142 | 2.1 | 2.3 | 13 |
| Seeds ^6^ | 1.4 | 1.3 | -10 | 0.5 | 0.5 | -16 | 0.7 | 0.9 | 26 |
| Milk | 248 | 235 | -5 | 280 | 254 | -9 | 159 | 151 | -5 |
| Yoghurt etc. | 101 | 101 | 0 | 148 | 144 | -3 | 24 | 19 | -21 |
| Cream, sour cream etc. | 70 | 66 | -5 | 43 | 37 | -14 | 126 | 120 | -5 |
| Cheese | 35 | 35 | -1 | 48 | 48 | 1 | 8.8 | 5.8 | -34 |
| Plant-based dairy alternatives | 1.0 | 0.9 | -5 | 1.3 | 2.5 | 92 | 0.8 | 0.8 | -1 |
| Meat total | 114 | 111 | -3 | 53 | 50 | -6 | 222 | 222 | 0 |
| Beef and lamb | 30 | 23 | -23 | 9.0 | 7.2 | -20 | 65 | 46 | -29 |
| Pork | 64 | 65 | 2 | 34 | 32 | -7 | 110 | 127 | 16 |
| Poultry | 21 | 23 | 8 | 10 | 11 | 12 | 47 | 49 | 3 |
| Egg | 39 | 38 | -1 | 37 | 34 | -8 | 27 | 23 | -15 |
| Fish, total ^7^ | 42 | 42 | -1 | 54 | 49 | -9 | 45 | 46 | 1 |
| Fats, plant-based | 26 | 27 | 3 | 34 | 38 | 11 | 16 | 17 | 4 |
| Fats, animal-based ^8^ | 31 | 30 | -6 | 27 | 19 | -29 | 14 | 12 | -10 |
| Discretionary foods and beverages | 108 | 121 | 12 | 174 | 188 | 8 | 69 | 71 | 3 |
| Condiments, seasoning, coffee and tea | 22 | 22 | 0 | 19 | 19 | 1 | 32 | 36 | 14 |

^1^ Combination of grains/flour and bread; ^2^ Includes mushrooms; ^3^ Includes berries, dried fruit and fruit juice; ^4^ Pulses purchased as a mix of cooked and dry pulses but are here expressed as dry weight; ^5^ e.g. soy-, pea-, and mycoprotein-based products, including tofu, plant-based nuggets, sausages etc.; ^6^ Does not include seeds in bread; ^7^ Meat and fish is predominantly raw but also contains limited amounts of processed products; ^8^ Includes fat-based products e.g. sauces and dressings

**References**

1. Meyers House of Food. Kurser for medarbejdere i Københavns Kommunes institutioner og tilbud (Courses for employees in Copenhagen Municipality’s institutions). https://maaltider.onlinebooq.dk/. Accessed 15 Aug 2023.

2. Organization WH. Protein and Amino acid Requirements in Human Nutrition. Report of a Joint WHO/FAO/UNU Expert Consultation. World Health Organization Technical Report Series. 2007.
